# IL-12 Inhibits Lipopolysaccharide Stimulated Osteoclastogenesis in Mice

**DOI:** 10.1155/2015/214878

**Published:** 2015-05-03

**Authors:** Masako Yoshimatsu, Hideki Kitaura, Yuji Fujimura, Haruka Kohara, Yukiko Morita, Noriaki Yoshida

**Affiliations:** ^1^Department of Orthodontics and Dentofacial Orthopedics, Nagasaki University Graduate School of Biomedical Sciences, 1-7-1 Sakamoto, Nagasaki 852-8588, Japan; ^2^Division of Orthodontics and Dentofacial Orthopedics, Department of Translational Medicine, Tohoku University Graduate School of Dentistry, 4-1 Seiryo-machi, Aoba-ku, Sendai 980-8575, Japan

## Abstract

Lipopolysaccharide (LPS) is related to osteoclastogenesis in osteolytic diseases. Interleukin- (IL-) 12 is an inflammatory cytokine that plays a critical role in host defense. In this study, we investigated the effects of IL-12 on LPS-induced osteoclastogenesis. LPS was administered with or without IL-12 into the supracalvariae of mice, and alterations in the calvarial suture were evaluated histochemically. The number of osteoclasts in the calvarial suture and the mRNA level of tartrate-resistant acid phosphatase (TRAP), an osteoclast marker, were lower in mice administered LPS with IL-12 than in mice administered LPS alone. The serum level of tartrate-resistant acid phosphatase 5b (TRACP 5b), a bone resorption marker, was also lower in mice administered LPS with IL-12 than in mice administered LPS alone. These results revealed that IL-12 might inhibit LPS-induced osteoclastogenesis and bone resorption. In TdT-mediated dUTP-biotin nick end-labeling (TUNEL) assays, apoptotic changes in cells were recognized in the calvarial suture in mice administered LPS with IL-12. Furthermore, the mRNA levels of both Fas and FasL were increased in mice administered LPS with IL-12. Taken together, the findings demonstrate that LPS-induced osteoclastogenesis is inhibited by IL-12 and that this might arise through apoptotic changes in osteoclastogenesis-related cells induced by Fas/FasL interactions.

## 1. Introduction

Lipopolysaccharide (LPS) is a large molecule consisting of a lipid and a polysaccharide joined by a covalent bond [1–4]. It is found in the cell walls of gram-negative bacteria and acts as an endotoxin. It induces a series of proinflammatory cytokines and results in the occurrence of strong immune responses. Osteolytic diseases such as periodontitis, osteomyelitis, and arthritis are related to LPS-induced immune reactions [5, 6]. Through binding to Toll-like receptor-4 on the surface of target cells, LPS induces the production of proinflammatory cytokines such as tumor necrosis factor- (TNF-) *α*, interleukin- (IL-) 1, and IL-6 [7–11].

Osteoclasts are multinucleated giant cells that originate from hematopoietic stem cells [12, 13]. They play important roles in bone resorption and remodeling in association with a series of transcription factors and cytokines. In osteolytic diseases, the formation and activity of osteoclasts are exceptionally stimulated. Receptor activator of necrosis factor-*κ*B ligand (RANKL) and macrophage colony-stimulating factor are known as major cytokines for osteoclastogenesis. TNF-*α* is also related to osteoclastogenesis [14–16].

IL-12 was reported to inhibit osteoclast formation in spleen cell cultures* in vitro* [17]. We previously found that IL-12 inhibited TNF-*α*-mediated osteoclastogenesis by inducing apoptosis of bone marrow cells* in vitro* [18, 19]. The induction of apoptosis was mediated by the interaction of TNF-*α*-induced Fas and IL-12-induced FasL [18, 19]. IL-12 was also shown to inhibit TNF-*α*-mediated osteoclastogenesis in the calvarial suture and during mechanical tooth movement* in vivo* [20, 21]. We reported that apoptotic changes were recognized histochemically when osteoclastogenesis was inhibited [20, 21]. Nagata et al. [22] confirmed that RANKL-induced osteoclastogenesis was inhibited by IL-12 and concluded that IL-12 might not be involved in cell death. Thus, several investigators have reported that IL-12 is related to inhibition of TNF-*α* or RANKL-induced osteoclastogenesis.

The aim of this study was to investigate the effects of IL-12 on bacterial LPS-induced osteoclastogenesis and bone resorption.

## 2. Materials and Methods

### 2.1. Mice and Reagents

Male 8-week-old C57BL6/J mice were purchased from SLC (Shizuoka, Japan) for use in this study. All animal care and experimental procedures were performed in accordance with the Guidelines for Animal Experimentation of Nagasaki University with approval of the Institutional Animal Care and Use Committee. LPS from* Escherichia coli* was purchased from Sigma (St. Louis, MO). Recombinant mouse IL-12 was purchased from R&D Systems (Minneapolis, MN).

### 2.2. LPS-Induced Osteoclastogenesis* In Vivo*

It has been reported that daily injections of LPS (100 *μ*g/day) for 5 days into the supracalvariae of mice were sufficient for osteoclast induction* in vivo* [23]. In this study, mice were divided into four groups and received daily injections of LPS alone (100 *μ*g/day), LPS with IL-12 (1.5 *μ*g/day), IL-12 alone (1.5 *μ*g/day), or phosphate-buffered saline (PBS) as a control. After 5 days of administration, the mice were euthanized and the calvariae were immediately removed. After overnight fixation with 4% paraformaldehyde at 4°C, the calvariae were demineralized in 10% EDTA for 4 days at 4°C. Paraffin-embedded samples were sectioned at 4 *μ*m. To observe osteoclasts, sections of the calvariae were deparaffinized, stained for tartrate-resistant acid phosphatase (TRAP), and counterstained with hematoxylin. The numbers of TRAP-positive cells in the calvarial suture were counted.

### 2.3. Microfocal Computed Tomography Assessment

The calvariae were fixed with paraformaldehyde and evaluated by microfocal computed tomography (RmCT; Rigaku, Tokyo, Japan) to clarify the bone resorption pits and calvarial suture expansion. Images of the calvariae were used for calculation of the radiolucent areas with ImageJ software (National Institutes of Health, Bethesda, MD). The relative values for the radiolucent areas in the groups were normalized by the value in the PBS group.

### 2.4. Serum Tartrate-Resistant Acid Phosphatase 5b (TRACP 5b) Assay

Serum was obtained from blood samples collected from the heart chambers under anesthesia. The serum levels of TRACP 5b were determined using a Mouse TRAP Assay Kit (IDS, Tyne and Wear, UK), in accordance with the manufacturer's protocol.

### 2.5. RNA Preparation and Real-Time RT-PCR Analysis

To isolate total RNA, mouse calvariae were frozen in liquid nitrogen, ground, and processed using an RNeasy Mini Kit (Qiagen, Valencia, CA). cDNA was synthesized from 1 *μ*g of total RNA using reverse transcriptase (Toyobo, Osaka, Japan) and random primers (Invitrogen) in a final volume of 20 *μ*L. The expression levels of TRAP, Fas, FasL, and TNF-*α* mRNAs were quantified by real-time RT-PCR using an Mx3000P/Mx3005P real-time PCR system (Stratagene, La Jolla, CA). Reactions were performed in a 25 *μ*L volume containing 2 *μ*L of cDNA, 12.5 *μ*L of SYBR Premix Ex Taq (Takara, Shiga, Japan), 10 *μ*M primers, and 0.5 *μ*L of ROX Reference Dye II. The primers used were as follows: GAPDH, 5′-ACCCAGAAGACTGTGGATGG-3′ and 5′-CACATTGGGGGTAGGAACAC-3′; TRAP, 5′-AACTTGCGACCATTGTTAGC-3′ and 5′-GGGGACCTTTCGTTGATGT-3′; Fas, 5′-TGGCAGAGGAGCCTAGTTGT-3′ and 5′-CACACCCAGGAACAGTCCTT-3′; FasL, 5′-ATCCCTCTGGAATGGGAAGA-3′ and 5′-CCATATCTGTCCAGTAGTGC-3′; TNF-*α*, 5′-CTGTAGCCCACGTCGTAGC-3′ and 5′-TTGAGATCCATGCCGTTG-3′. The cycling conditions were as follows: initial denaturation at 95°C for 10 s; 45 cycles of amplification, each comprising a denaturation step at 95°C for 5 s and an annealing step at 60°C for 20 s. The relative expression levels of TRAP, Fas, FasL, and TNF-*α* mRNAs were normalized by the corresponding expression levels of GAPDH mRNA.

### 2.6. Apoptosis Detection by the TdT-Mediated dUTP-Biotin Nick End-Labeling (TUNEL) Assay

An ApopTag Peroxidase* In Situ* Apoptosis Detection Kit (Chemicon International, Temecula, CA) was used for TUNEL staining. Deparaffinized sections were pretreated with 20 *μ*g/mL proteinase K for 15 min and then incubated with 3% hydrogen peroxide for 5 min at room temperature to quench endogenous peroxidase activity. Next, the sections were sequentially incubated with TdT enzyme for 1 h at 37°C and antidigoxigenin peroxidase, followed by development with a diaminobenzidine peroxidase substrate. The sections were counterstained with methyl green.

### 2.7. Statistical Analysis

All data are presented as means ± SD. Statistical analyses were performed using Scheffe's *F* tests. Differences were considered significant when *P* < 0.05.

## 3. Results

### 3.1. IL-12 Inhibits LPS-Induced Osteoclastogenesis in the Mouse Calvariae

**Figure 1:**
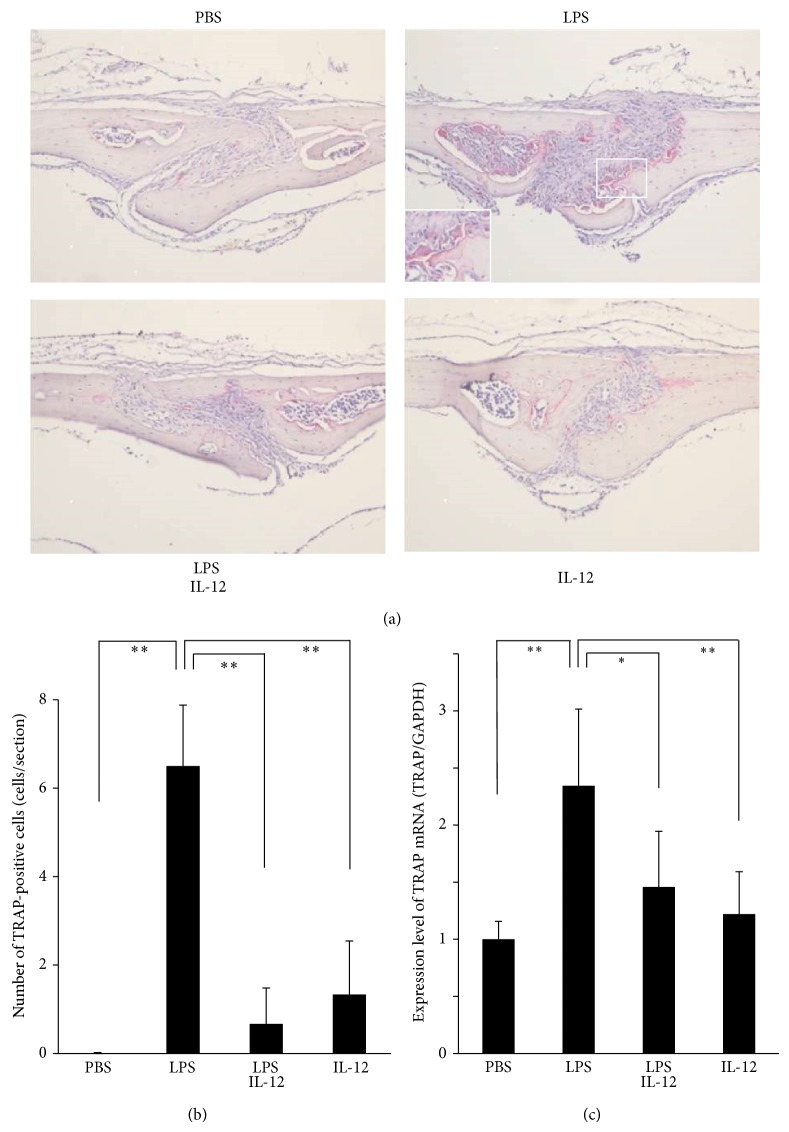
Osteoclastogenesis in the calvarial suture. (a) Histological sections of calvariae excised from mice after daily supracalvarial administrations of PBS, LPS alone (100 *μ*g/day), LPS with IL-12 (1.5 *μ*g/day), or IL-12 alone for 5 days. The sections were stained for TRAP activity. (b) Numbers of TRAP-positive cells in the calvarial suture. (c) TRAP mRNA levels in the mouse calvariae detected by real-time RT-PCR. Total RNA was isolated from the mouse calvariae after daily supracalvarial injections of PBS, LPS, LPS with IL-12, or IL-12 for 5 days. The TRAP mRNA levels were normalized by the corresponding GAPDH mRNA levels. Results are expressed as means ± SD (*N* = 6; ^*^ *P* < 0.05, ^**^ *P* < 0.01). Differences were detected using Scheffe's *F* tests.

To analyze the effects of IL-12 on LPS-induced osteoclastogenesis* in vivo*, TRAP staining of paraffin-embedded sections was performed after LPS was administered with or without IL-12 into mouse supracalvariae. In the LPS alone group, many TRAP-positive cells were observed in the calvarial suture (Figure 1(a)). On the other hand, there were few TRAP-positive cells in the LPS with IL-12 group. The number of TRAP-positive cells, counted as osteoclasts, was significantly reduced in the LPS with IL-12 group compared with the LPS alone group (Figure 1(b)). The levels of TRAP mRNA in the calvariae were determined by real-time RT-PCR. The expression of TRAP was significantly higher in the LPS alone group than in the PBS group. Furthermore, the level of TRAP was significantly decreased in the LPS with IL-12 group compared with the LPS alone group (Figure 1(c)).

### 3.2. IL-12 Inhibits LPS-Induced Bone Resorption in the Mouse Calvariae

**Figure 2:**
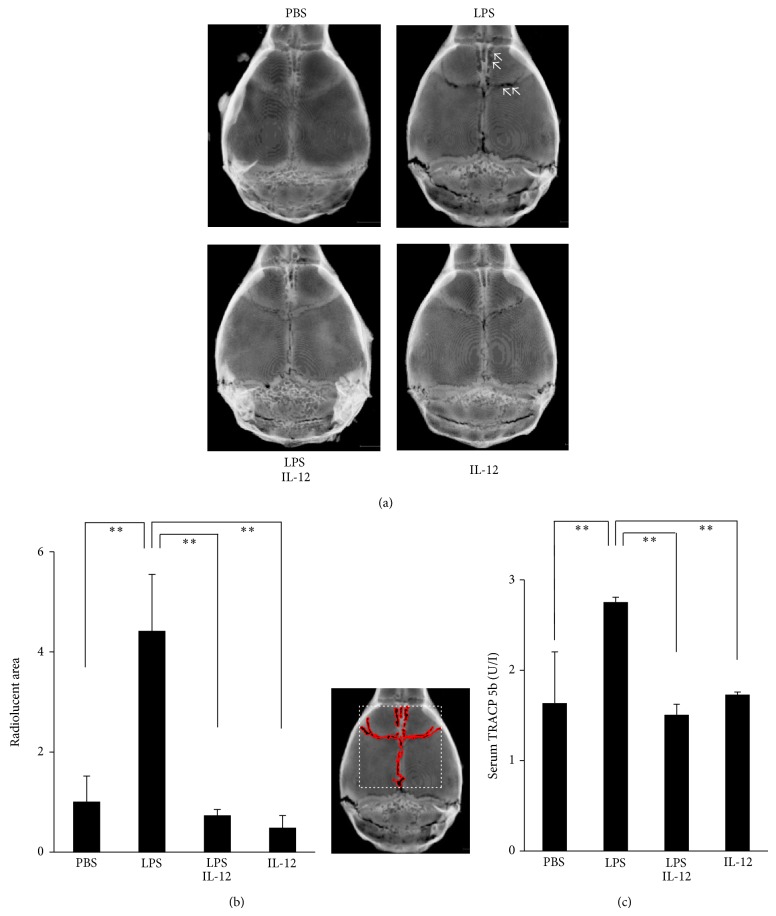
Radiolucent areas in the mouse calvariae. (a) Microfocal computed tomography reconstructed images of calvariae. Images of calvariae excised from mice after daily supracalvarial administrations of PBS, LPS alone (100 *μ*g/day), LPS with IL-12 (1.5 *μ*g/day), or IL-12 alone for 5 days. The arrows show the resorption lacunae. (b) Evaluation of the radiolucent areas on the calvariae. The radiolucent areas in the dotted boxed area shown in the right side of the graph were calculated. The red areas indicate the radiolucent areas. The relative values of the radiolucent areas in the groups were normalized by the corresponding values in the PBS group. (c) Serum levels of TRACP 5b. Circulating TRACP 5b levels were determined by ELISA. Results are expressed as means ± SD (*N* = 9; ^**^ *P* < 0.01). Differences were detected using Scheffe's *F* tests.

To investigate the effects of bone resorption by IL-12 on LPS-induced osteoclastogenesis, the radiolucent areas in calvariae were observed by RmCT after LPS was administered with or without IL-12 into mouse supracalvariae. In the LPS alone group, the radiolucent area was expanded on the mouse calvariae (Figures 2(a) and 2(b)). On the other hand, in the LPS with IL-12 group, the radiolucent area was decreased compared with the LPS alone group. Furthermore, the serum TRACP 5b levels were lower in the LPS with IL-12 group than in the LPS alone group (Figure 2(c)).

### 3.3. Administration of LPS with IL-12 Induces Apoptosis in the Mouse Calvariae

**Figure 3:**
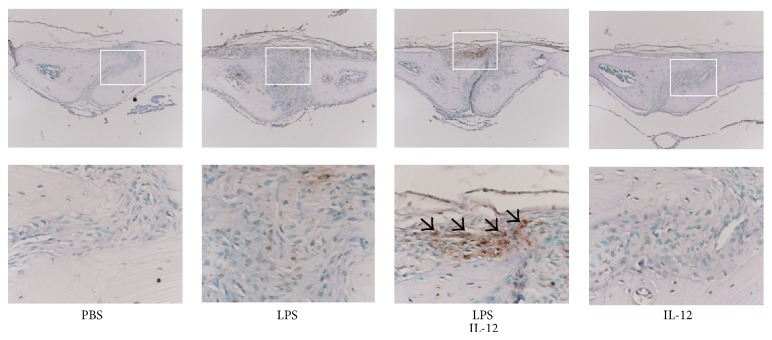
Induction of apoptosis by LPS and IL-12 in the mouse calvariae. Histological sections of calvariae excised from mice after daily supracalvarial injections of PBS, LPS alone (100 *μ*g/day), LPS with IL-12, or LPS alone (1.5 *μ*g/day) for 5 days were subjected to TUNEL staining to detect apoptotic cells. The lower panels show high-magnification images of the boxed areas in the upper panels. Apoptotic cells are indicated by arrows.

IL-12 was previously reported to induce apoptosis of osteoclast precursor cells in TNF-*α*-induced osteoclast formation and inhibit osteoclast formation in TNF-*α*-administered mice [18, 20]. Because osteoclastogenesis was decreased in the LPS with IL-12 group, we performed histological examinations with TUNEL staining to determine whether induction of apoptosis occurred in the calvarial suture. In the LPS alone group, few TUNEL-positive cells were observed. In comparison, many TUNEL-positive cells were observed in the LPS with IL-12 group (Figure 3).

### 3.4. Administration of LPS and IL-12 Affects Expression of Fas and FasL

**Figure 4:**
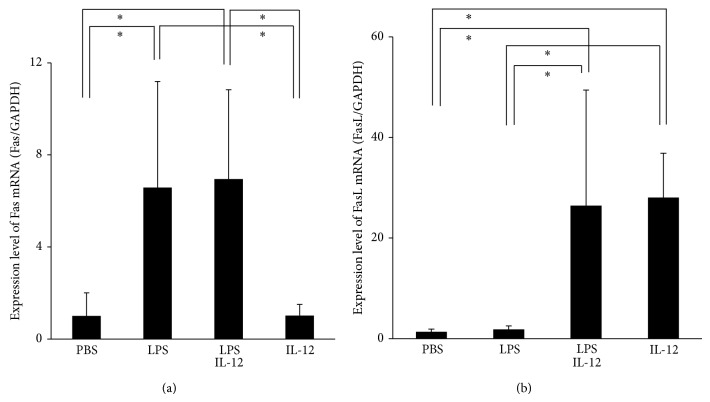
Fas and FasL mRNA levels in the mouse calvariae detected by real-time RT-PCR. Total RNA was isolated from mice calvariae after daily supracalvarial injections of PBS, LPS alone (100 *μ*g/day), LPS with IL-12 (1.5 *μ*g/day), or IL-12 alone for 5 days. (a) Fas mRNA levels. (b) FasL mRNA levels. The mRNA levels of Fas and FasL were normalized by the corresponding GAPDH mRNA levels. Results are expressed as means ± SD (*N* = 6; ^*^ *P* < 0.05). Differences were detected using Scheffe's *F* tests.

To elucidate how apoptosis was induced in the mouse calvariae, the expression levels of Fas and FasL mRNAs were examined by real-time RT-PCR. The expression levels of Fas mRNA were significantly higher in the LPS alone and LPS with IL-12 groups than in the PBS group (Figure 4(a)). In addition, the expression levels of FasL mRNA were significantly higher in the LPS alone and LPS with IL-12 groups than in the PBS group (Figure 4(b)).

### 3.5. LPS Induces Expression of TNF-*α* in the Mouse Calvariae

**Figure 5:**
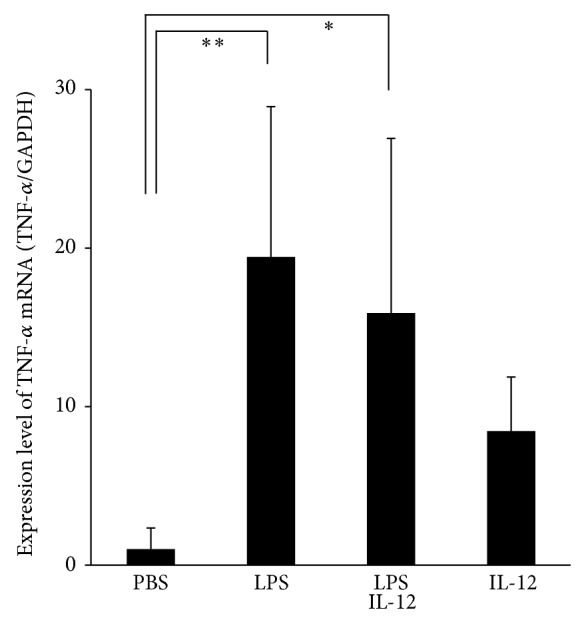
TNF-*α* mRNA levels in the mouse calvariae detected by real-time RT-PCR. Total RNA was isolated from mice calvariae after daily supracalvarial injections of PBS, LPS alone (100 *μ*g/day), LPS with IL-12 (1.5 *μ*g/day), or IL-12 alone for 5 days. The mRNA levels of TNF-*α* were normalized by the corresponding GAPDH mRNA levels. Results are expressed as means ± SD (*N* = 6; ^*^ *P* < 0.05, ^**^ *P* < 0.01). Differences were detected using Scheffe's *F* tests.

The levels of TNF-*α* mRNA were examined by real-time RT-PCR to determine how apoptotic changes were induced in the calvarial suture. It is important to assess the levels of TNF-*α*, because it is a key factor for osteoclastogenesis. The results indicated that the levels of TNF-*α* mRNA were increased in the LPS alone and LPS with IL-12 groups compared with the PBS group (Figure 5).

## 4. Discussion

In this study, we have demonstrated the effects of IL-12 on LPS-induced osteoclastogenesis in mouse calvariae* in vivo*. Previously, a number of investigators have examined LPS-mediated osteoclastogenesis [7, 23–25]. Among these reports, there were two* in vivo* studies in which LPS was administered especially into calvariae [23, 25]. The protocol, dose, and days of LPS administration were based on these reports. As shown by RmCT and histological images, daily injections of LPS (100 *μ*g/day) for 5 days into the supracalvariae were sufficient for osteoclast induction in calvariae in this study. Loss of bone and expansion of the calvarial suture were observed after LPS administration.

Although a number of studies have investigated the functions of IL-12, there are few studies related to osteoclastogenesis. IL-12 is mainly produced by macrophages, dendritic cells, and B cells and induces cytotoxic properties of T cells and NK cells [26, 27]. One of these studies further showed that IL-12 plays a pivotal role in controlling innate and adaptive immunity against a variety of infections [26]. IL-12 particularly induces the production of interferon- (IFN-) *γ*, a potent activator of antimicrobial functions and tumor control, by T cells and NK cells. IL-12 can also induce the differentiation and proliferation of T-helper 1 (Th1) cells from Th0 cells [26]. Kerkar et al. [28] indicated that IL-12 triggers myeloid-derived cells sensitized for tumor destruction, while Eisenring et al. [29] showed that IL-12 induces tumor suppression by stimulating a subset of NKp46^+^ lymphoid tissue-inducer cells. Thus, IL-12 is related to various immunological responses. Although some investigators have examined the expression of IL-12 during LPS-induced osteoclastogenesis [30, 31], no studies have clarified how IL-12 affects LPS-induced osteoclastogenesis. Therefore, we investigated the effects of IL-12 on LPS-induced osteoclastogenesis. LPS was administered with or without IL-12 into the mouse supracalvariae to evaluate how IL-12 affects LPS-induced osteoclastogenesis* in vivo*. In the LPS alone group, many osteoclasts and bone destruction spots were recognized in the calvarial suture. On the other hand, in the LPS with IL-12 group, osteoclasts and bone destruction spots were decreased. The levels of TRAP mRNA in the mouse calvariae were also decreased in the LPS with IL-12 group compared with the LPS alone group. These results suggested that IL-12 can inhibit LPS-mediated bone resorption.

LPS is known to induce proinflammatory cytokines [7–11]. TNF-*α*, a proinflammatory cytokine, is related to osteoclastogenesis [14–16]. In the present study, when LPS was administered alone into the mouse calvariae, expression of TNF-*α* was increased at the mRNA level. TNF-*α* might be related to the loss of calvarial bone when LPS was administered alone. TNF-*α* was also increased when IL-12 was administered with LPS, compared with administration of PBS alone. Previous reports have shown that TNF-*α*-induced osteoclastogenesis was inhibited by IL-12* in vitro *and* in vivo* [18–21, 32, 33]. They concluded that osteoclastogenesis might be inhibited by apoptotic changes in osteoclast precursor cells and might be mediated by interactions between TNF-*α*-upregulated Fas and IL-12-upregulated FasL. In this study, the expression level of Fas was increased in the LPS alone and LPS with IL-12 administered groups, and FasL was increased in the LPS with IL-12 and IL-12 alone administered groups. These findings indicated that the apoptotic changes in calvarial cells might be caused by interactions of TNF-*α* (induced by LPS) induced Fas and IL-12-induced FasL. Zhang et al. [34] showed that LPS increased Fas expression on memory B (mB) cells and caused mB cell apoptosis through the Fas/FasL pathway. In this study, LPS might, in part, be directly related to Fas/FasL interactions. Yim et al. [35] reported that IL-12 has the ability to induce macrophage apoptosis by IFN-*γ*-induced nitric oxide synthesis. IL-12 is able to induce tumor apoptosis in mouse hepatocellular carcinoma* in vivo* [36]. The IL-12-related apoptotic changes in tumor cells are mediated by T lymphocytes, NK cells, and NKT cells. The authors mentioned the involvement of IFN-*γ* in their report. Nagata et al. [22] previously demonstrated that RANKL-induced osteoclastogenesis is possibly inhibited by IFN-*γ*, which was induced by IL-12* in vitro*. Thus, IFN-*γ* is possibly also associated with the IL-12-mediated inhibition of osteoclastogenesis. However, IFN-*γ* was not increased at the mRNA level in the groups administered IL-12 in this study (data not shown). Therefore, the inhibition of osteoclastogenesis observed when IL-12 was administered with LPS in this study might not be related to IFN-*γ*. Yang et al. [37] reported that LPS induces osteoclastogenesis through the induction of RANKL expression in osteoblasts. It means that RANKL is related to LPS-induced osteoclastogenesis. It has also been reported previously that IL-12 inhibited RANKL-induced osteoclastogenesis via a nonapoptotic pathway [22]. Therefore, it is possible that IL-12 may also inhibit osteoclastogenesis mediated by LPS-induced RANKL expression. It is possible that several complicating factors could be responsible for the occurrence of apoptosis in osteoclastogenesis-related cells. Additionally, the identity of the apoptotic cells is also not clear at this stage. Further studies are required to clarify these points.

In summary, our study has demonstrated that IL-12 inhibits LPS-induced osteoclastogenesis* in vivo*. Furthermore, the mRNA levels of Fas and FasL were both increased in mice administered LPS with IL-12 and it might lead to apoptotic changes in osteoclastogenesis-related cells through Fas/FasL interactions.
